# Reducing the Training Samples for Damage Detection of Existing Buildings through Self-Space Approximation Techniques

**DOI:** 10.3390/s21217155

**Published:** 2021-10-28

**Authors:** Alberto Barontini, Maria Giovanna Masciotta, Paulo Amado-Mendes, Luís F. Ramos, Paulo B. Lourenço

**Affiliations:** 1 Department of Civil Engineering, ISISE, University of Minho, 4800-058 Guimarães, Portugal; albe.barontini@gmail.com (A.B.); lramos@civil.uminho.pt (L.F.R.); 2 Department of Engineering and Geology, University G. d’Annunzio of Chieti-Pescara, 65127 Pescara, Italy; g.masciotta@unich.it; 3 Department of Civil Engineering, ISISE, University of Coimbra, 3030-788 Coimbra, Portugal; pamendes@dec.uc.pt

**Keywords:** structural health monitoring, damage detection, historical buildings, negative selection algorithm

## Abstract

Data-driven methodologies are among the most effective tools for damage detection of complex existing buildings, such as heritage structures. Indeed, the historical evolution and actual behaviour of these assets are often unknown, no physical models are available, and the assessment must be performed only based on the tracking of a set of damage-sensitive features. Selecting the most representative state indicators to monitor and sampling them with an adequate number of records are therefore essential tasks to guarantee the successful performance of the damage detection strategy. Despite their relevance, these aspects have been frequently taken for granted and little attention has been paid to them by the scientific community working in the field of Structural Health Monitoring. The present paper aims to fill this gap by proposing a multistep strategy to drive the selection of meaningful pairs of correlated features in order to support the damage detection as a one-class classification problem. Numerical methods to reduce the number of necessary acquisitions and estimate the performance of approximation techniques are also provided. The analyses carried out to test and validate the proposed strategy exploit a dense dataset collected during the long-term monitoring of an outstanding heritage structure, i.e., the Church of ‘Santa Maria de Belém’ in Lisbon.

## 1. Introduction

Thanks to the latest improvements in sensing architecture (e.g., reduced size and cost, increased reliability and ruggedness) and in computational strategies (e.g., faster and reliable data fusion, machine learning, and optimisation algorithms), nowadays Structural Health Monitoring (SHM) systems can be composed of large quantities of sensors of different typologies and are capable of acquiring data with distinct sampling rate and formats. These peculiarities make SHM-based methodologies very attractive for stakeholders, allowing to collect—either directly or indirectly—a wide range of damage-related observable quantities (e.g., cracks width, tilting, accelerations, velocities, etc.) and to integrate the information content of each of them, by combining their sensitivity to damage with their precision as global or local indicators. However, the result is a large amount of information, easily up to terabytes of unstructured or semi-structured data per week [1], potentially able to reduce the uncertainty, but extremely more demanding in terms of data management and interrogation [2]. The complexity and the volume of this data flow is comparable to the so-called “Big Data”, which refers to datasets that are almost unmanageable and unprocessable with traditional approaches and tools [3,4,5,6]. Thus, to ensure the effectiveness of the structural monitoring as well as the interest of the stakeholders, the SHM system should be designed in order to deliver and archive only the minimum amount of necessary information, according to a process that can be referred to as feature selection. This consists in the identification of the most significant Quantities of Interest (QoI) for the structure—which might vary depending on the specific goal of the monitoring—and in the definition of the best way to estimate them with enough accuracy. Not all the damage-sensitive QoI, in fact, can be directly measured. In some cases, they are extracted from a related observable quantity. The extraction is part of the data pre-processing stage together with other processes such as data cleansing, data reduction, and data fusion. The aim of such techniques is to correct or reject noisy or contaminated data and to merge information from different sources, producing a new dataset with comparable reliability and information content, but reduced dimensionality and uncertainty. QoI uncertainty and variability also depend on non-damage-related factors. For instance, it is well-known that environmental and operational variations (EOVs) may sensibly affect the properties of a structural system (e.g., in terms of mass, stiffness, and stress level), resulting in changes of its dynamic response. A brief state of the art on this topic is provided in Section 2.1. It follows that the feature selection task is extremely problem-specific and must be tailored to the peculiar characteristics of both the investigated system and the environment to which it is exposed.

Applying a feature selection strategy to a large dataset of a well-known structure is relatively easy but investigating the structural fitness of a system that is completely unknown, and whose behaviour is affected by many uncertainties, is quite challenging. This situation is common when dealing with large historical constructions as in most cases they were built in different phases, with various materials and structural elements, and are therefore characterised by different states of degradation and accumulated damage. Past interventions and unknown extreme loadings might have significantly jeopardized the conditions of such buildings, leading to a behaviour that can be complex to predict and interpret. Under these circumstances, the development of a reliable model for health assessment needs a representative baseline of feature value distributions for different operational and environmental conditions that may require a long preliminary monitoring campaign. Reducing the size of the sample set necessary to develop and train such models means reducing the cost, time, and computational burden associated to them. This is particularly beneficial when the preliminary monitoring stage is carried out using a continuous monitoring system (i.e., by installing permanent sensors across the structure), but it is even more important when the SHM system is deployed and dismantled after every record, since it allows to plan in advance the acquisitions.

This paper originates from the research framework outlined above and aims at tracing a cost-effective feature selection strategy for SHM of historical buildings. The proposed approach is meant to support the damage detection task intended as one-class classification problem, which consists in the assessment of the state of a system as either safe or unsafe, based on a set of features measured repeatedly over time. The classifier is a function trained through a dataset from the reference state of the system. Among the algorithms suitable to address such problems, the authors have recently investigated the applicability of Negative Selection Algorithms (NSAs), developing a deterministic generation based version that analyses the evolution of pairs of features at the same time [7,8,9]. The feature selection strategy that will be described in Section 2 is tailored to this new version of the NSA and it is based on the pairwise correlation of structural properties between themselves and with non-structural factors. Moreover, different procedures to reduce the number of acquisitions needed to achieve a good approximation of the system behaviour in the reference condition are presented in the same section and appropriate codes developed for this purpose are exploited. It is worth noting that such methodologies, although rooted in the same NSA mode of operation, can be beneficial to any anomaly detection strategy, since the result is a set of points in the feature space which can be employed to approximate the possible response of any system in its reference condition and to train any data-driven algorithm for damage detection. To establish whether or not the sample set is large enough, irrespective of the goals of the monitoring, specific performance metrics are tested in Section 3, to rate the approximation. Given the necessity of a large validating dataset, the tests presented hereafter are carried out by using quite a unique data set collected from the long-term monitoring of the Church of the Jerónimos Monastery in Lisbon [10,11], which have also benchmarked a previous experience of the authors of this work [8]. An extensive discussion of the main results is provided in Section 4 with focus on the integration of all the proposed strategies into a single methodology and on the analysis of the potential advantages for stakeholders operating in the civil engineering SHM field. Finally, the conclusions are drawn in Section 5.

## 2. Interpreting the Variability of The Structural Response to Support Damage Detection

### 2.1. Effects of EOVs on Vibration Properties

A growing body of evidence about the effects of EOVs on buildings’ vibration properties has been published since the late 1980’s, especially in bridge monitoring. In this regard, relevant examples ranging from short-span [12,13,14,15] to long-span bridges [16,17,18] exist. More recently, similar studies have been carried out on large-scale structures, such as stadiums or tall buildings [19,20,21]. The long-term monitoring of monuments confirmed the influence of environmental conditions for this type of structures as well, and various examples have been reported from several countries [11,22,23,24,25,26,27,28,29,30,31]. Although the majority of them consist of stone/brick masonry buildings, earthen structures are not exempt from these effects either [32,33].

Typical environmental conditions that affect the behaviour of structures and infrastructure systems are [34]: aerodynamic and hydrodynamic loadings; radiation environment; temperature, moisture, and humidity levels. As regards operational conditions, they usually depend on the live loads, which can be static or dynamic (e.g., traffic-induced loading). Civil engineering systems related to significant mechanical processes, such as those hosting heavy machinery, power plants, oil extraction pumps, etc., also undergo different conditions due to power fluctuation, start-up and shut-down transients, moving parts manoeuvres, changing mass loading (e.g., fuel levels in tanks, payloads of vehicles operating over the system, etc.) [34]. Environmental conditions can affect structures, irrespective of their nature, through different processes, such as the thermal expansion and contraction of the material (eventually modifying boundary conditions and causing cracks to close and open), the stiffness variation, the stress (re)distribution, the appearance of nonlinear effects and friction between elements, the variation of water content, and freezing and thaw phenomena [15,35,36,37,38]. Such processes induce fluctuations in the global vibration properties of the system, namely natural frequencies and structural damping, whereas modal displacements are not as sensitive as long as the physical properties are altered uniformly and the bearing conditions are not modified [36,39]. However, local nonuniform variations of the physical properties, induced by EOVs, may also significantly affect the mode shapes and, in the case of temperature effects, lead to seasonal cyclic trends [40]. The combination of all the effects results in different trends of the structure-specific vibration modes [19,41], thus distinguishing the contribution of each single factor, based on the time histories, is almost impossible.

Masonry structures exhibit a peculiar behaviour opposite to concrete and metal systems, since the frequencies tend to increase linearly with the temperature. This has been explained as an effect of the superficial cracks and discontinuities that close due to the thermal expansion of the material. Still, for very low temperatures, the frequencies increase inversely due to the closure of the cracks induced by the freezing of the system [28,29,42,43,44]. This phenomenon in some cases starts for temperature above 0 °C, determining either a non-linear correlation or a bilinear correlation with a knee. Other local phenomena can reduce the structural stiffness, and in turn the frequencies, with increasing temperature, including a progressive out of plumb or the slackening of ties [42,44,45]. A few studies also investigated the effect of relative humidity and wind speed on the structural behaviour. Regarding the former, it has been demonstrated that increasing relative humidity, followed by water absorption, can cause downshifts in the natural frequencies of masonry structures due to the mass increment [30,45]. As for the latter, it has been shown that increasing wind actions can induce large tensile stresses in tall masonry buildings, which are likely to reduce their stiffness, thus their frequencies [46]. In some cases the environmental effects present a time lag as compared to the monitored quantity, that varies based on the physics of the phenomenon [32,33].

The problem of developing a model able to explain and forecast the ambient-induced changes of the modal properties of a structural system is particularly relevant for damage detection purposes since the same modal properties are commonly adopted as damage indicators. Indeed, research experiences demonstrated that frequency shifts due to low-to-medium damage extents can be very small and easily masked by larger variations induced by environmental conditions [15]. An extensive discussion on possible strategies to filter-out non-damage-induced variations falls outside the scope of the paper, hence interested readers can refer to [17,34,35,47,48,49,50].

### 2.2. Vibration-Based Damage Detection

In the context of SHM, damage detection can be formulated as a pattern recognition problem addressed through machine learning algorithms [51]. In many civil engineering applications, the lack of information on the system response in a condition that differs from the reference one does reduce the damage detection problem to one-class classification [7], the goal being to associate the measured features of the system to one of the two possible condition states, e.g., safe/undamaged or unsafe/damaged, by training on baseline samples belonging to the safe state only. Machine learning algorithms may require a rescaling of the analysed features to a predefined range [52], for instance to train and implement a classifier within a unitary feature space, $U={\left[0,\;1\right]}^{n}$, where n is the number of features analysed (in the case of vibration-based damage detection, the features are derived from vibration signals).

U consists of two complementary subsets, called self, S, and non-self, NS, such that:

(1) S∪NS=U    S∩NS=∅

In a two-dimensional feature space, the self-space S is the region composed of all the possible pairs of feature values belonging to the system in healthy condition. It has potentially infinite elements due to the fact that EOVs and data noise induce shifts in the feature values even without a change of the system state from healthy to damaged. By contrast, the non-self-space NS is a region of the feature space gathering pairs of feature values which belong to another, potentially damaged, condition of the system. The previously introduced NSA performs the classification in terms of distance between new features and detectors, which approximate the non-self-space. The detectors are generated and trained against a set of representative self-samples $S^{\prime }$. This set approximates the whole self-space. Based on $S^{\prime }$, in fact, a self-representation space $\hat{S}$ is modelled relying on the concept of closeness, introduced through the self-radius $r_{self}\left(s^{i}\right)$, namely all the points x of the feature space falling within a distance of $r_{self}\left(s^{i}\right)$ around the i-th self-sample $s^{i}$ are considered self:

(2) $$\hat{S}=\left\{x\in U|\exists s^{i}\in S^{\prime },\|s^{i}-x\|\leq r_{self}\left(s^{i}\right)\right\}$$

**Figure 1:**
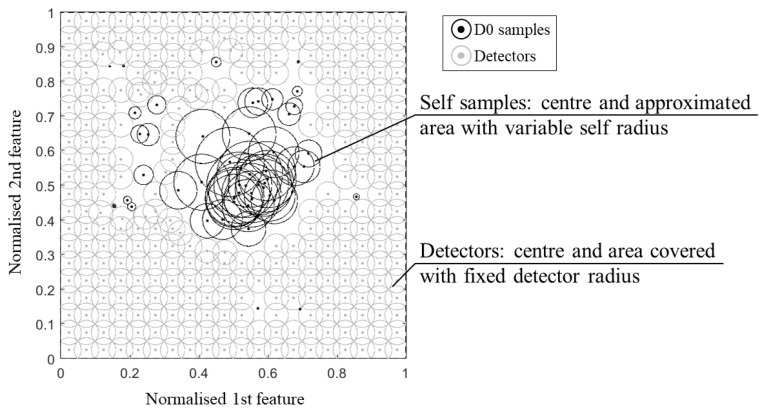
Example of detector distribution within a two-dimensional feature space after rejecting the detectors that match with the training self-samples, here considered with a variable self-radius.

It is evident that the self-radius is critical for the performance of the classification, especially for non-linear sample sets. A small radius can increase the false alarm rate (detectors might fall in the holes within the self-samples), whereas a big radius decreases the detection rate (the approximate self-space is larger than the actual, including portions of non-self-space). In general, the definition of the radius is related to the problem of boundary invasion (approximate self-space invading the non-self-space and/or detector set invading the self-space). However, in most of the NSAs, this is a constant value whose definition is not properly addressed. Only a few authors faced the self-representation by using a variable self-radius, according to the statistics of the sample distribution. This allows us not only to provide a better approximation of the self-space but also to reduce the number of samples required and their overlapping. In Figure 1, an example of two-dimensional feature space, where the self-samples $S^{\prime }$ with variable radius, are used to censor the detectors by rejecting the ones that match with the approximate self-space. New samples falling within the portion uncovered by the detector circles are not detected, thus, the system is classified as undamaged.

The use of statistical tools is due to the fact that the self-samples usually do not have a uniform distribution; they rather fall into a relatively stable range with a high overlapping of the samples in dense areas. Therefore, a bigger radius should be set for the samples of the dense region and a smaller radius should be considered in the case of samples falling in the sparse region. Consequently, less samples with a bigger radius will cover more space, allowing to discard the redundant overlapping samples. Among the algorithms for the improved self-representation, some methodologies able to deal with such a scalability problem have been identified [53,54,55]. One of them has been already applied by the authors in [9] to elaborate an enhanced version of NSA for the damage detection of a scaled masonry arch tested in laboratory conditions, pointing out the possible advantages offered by the proposed approach but also highlighting the need for further analyses on full-scale structures exposed to real environmental and operational conditions. The present research work provides a contribution to this latter point.

### 2.3. Feature Selection Strategy

**Figure 2:**
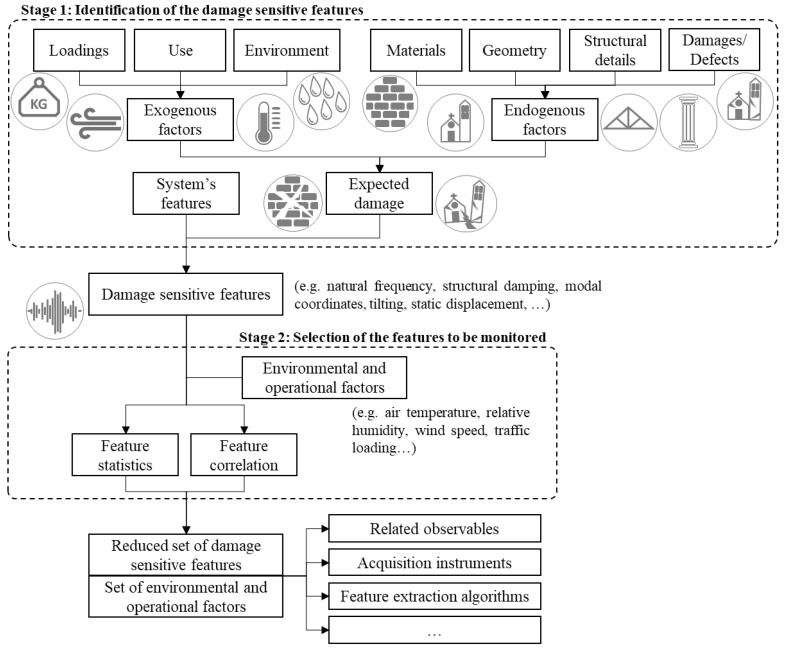
Flowchart of the feature selection strategy.

Feature selection for SHM is the process that aims at identifying the QoI, namely the system features to be monitored and the exogenous factors whose effect must be filtered out, to support damage detection. The strategy analysed in the present paper is composed of two stages, as depicted in Figure 2. A preliminary study is first performed to identify: (1) the endogenous factors, namely the sources of vulnerability of the system, its material and geometrical properties, and the aspects associated with its condition state (e.g., material defects, current damages, or evidence of previous damages); (2) the exogenous factors, such as hazards, peculiar operational conditions, loading, and environmental factors.

Engineering judgment plays a relevant role in performing this task, and it should always be supported by the knowledge acquired on similar cases, documentation, surveys, inspections, sampling, experimental tests, and analytical and numerical simulations. It is worth noting that these are typical sources for structural assessment and retrofitting, as recommended by codes and standards, as the Eurocode 8 part 3 [56], the International Existing Building Code [57], the ASCE/SEI Standard 41-06 [58], or the ISO 13822 [59], among others. It follows that this stage of SHM can be framed into the actual methodology to evaluate existing buildings, without additional costs, establishing a profitable link between punctual assessment and long-term, informed, smart management of the assets. By means of these sources, the aim of feature selection is the definition of future damage scenarios under possible hazardous events, paying attention to the potential development of ongoing failure mechanisms or degradation/alteration patterns. A set of endogenous features representative of the system state are then selected according to their sensitivity to the expected damage scenarios. The features selected according to these criteria will likely own a large information content and could be good targets for the monitoring system to readily warn in case of anomalous behaviour.

In the second stage, the non-damage-induced variability of the system features is analysed by investigating their normal range of variation against changing environmental and operational conditions and their correlation with them. This process helps defining which non-structural features should be monitored, if required, and which damage-sensitive features should be rejected from the list identified in the first stage, due to their excessive sensitivity to other parameters that might hide damage-induced variations. Typical statistical measures of central tendency (e.g., mean and median), of dispersion (e.g., standard deviation and interquartile range) and shape (e.g., skewness and kurtosis) are used to analyse the features value distribution. The correlation is qualitatively assessed by plotting graphs, such as the seasonal trends and the scatter plots, and quantitatively defined through indices such as the Pearson correlation coefficient. Whenever the relationship between the variables looks strongly non-linear, other metrics can be used as, for instance, the Spearman’s rank correlation coefficient.

The goal is the identification of a pair of properties forming a self-space with low scatter. It is assumed that, due to the good correlation between the features, when a new acquisition provides a point that falls outside the self-space, the system is likely to present an anomalous and potentially damaged behaviour. Moreover, it is assumed that the better the correlation, the smaller is the damage extent needed to push the value outside the identified self-space. The effective damage sensitivity of the features can be estimated by predicting the structural response against possible damage scenarios through experimental or numerical simulations and then comparing the trend of the tracked features with their reference baseline. Although, it must be stressed that predictions require either the support of accurate models that are not always available or the possibility to carry out experimental campaigns on accurate replica that are extremely expensive and often unfeasible, turning this approach potentially misleading if the sources of uncertainties are not correctly taken into account. To overcome this problem, it would be beneficial to use the same classification algorithm to train and implement more classifiers in parallel operating in distinct feature spaces (see example in Section 3.1) in order to take advantage of their different sensitivity to various damage scenarios.

The identification of the final set of features to be monitored implies the definition of the related observables, the characteristics of the acquisition system, the algorithms for feature extraction from the observables and other specific requirements of the monitoring process.

### 2.4. Self-Space Approximation

**Figure 3:**
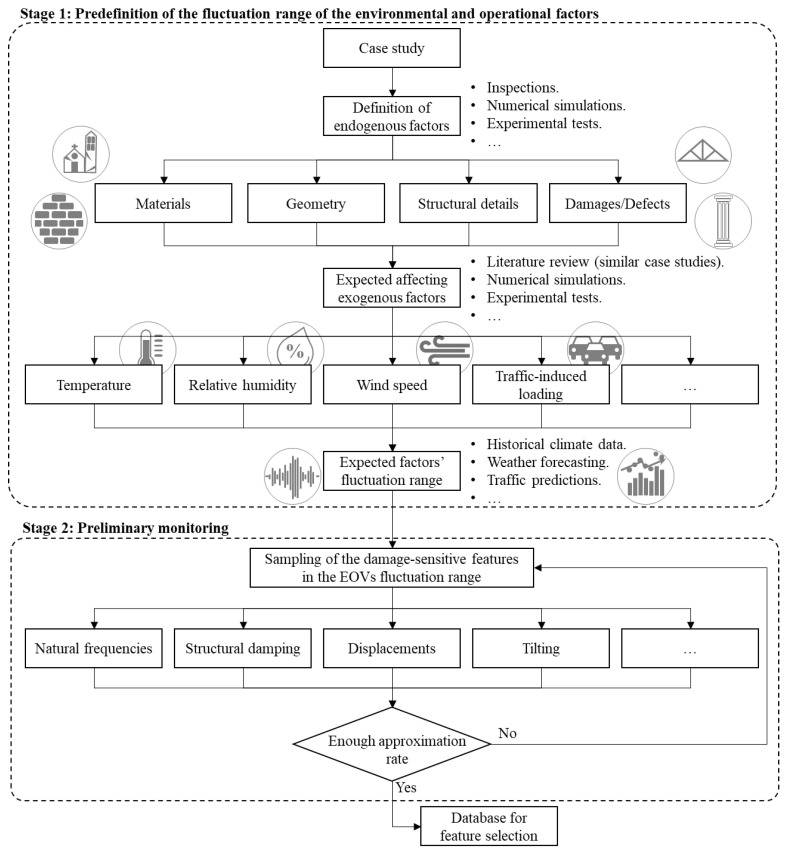
Flowchart of the self-space approximation strategy.

As previously mentioned, the feature selection strategy aims at the identification of the most representative features to be monitored based on the analysis of the self-region in the feature space, especially in terms of values distribution. Still, the real challenge is to perform this stage resorting to a reduced number of samples, in order to decrease the computational load and, most importantly, the time and costs associated with the acquisition. To be successful, this reduced baseline must be an unbiased sample set closely indicative of the feature value distributions, namely it must be representative of the feature fluctuations in the reference state, due to non-damage factors. Since various system’s features present daily and seasonal trends, in many geographical areas, a proper investigation to set baseline information would require at least a six-month time horizon; commonly, 18 months are recommended. Still, a continuous monitoring over this time frame is not necessary if a good approximation based on a reduced sample set is defined, with enormous advantages in terms of costs and data overload. In this regard, a set of self-approximation strategies are discussed hereafter. A generic flowchart is reported in Figure 3. Such strategies are usually divided in two stages. The former stage aims at the definition of the expected fluctuation range for the operational and environmental factors that are likely to affect most the system structural response in terms of damage-sensitive features. The latter stage consists of the preliminary monitoring of the damage-sensitive features under different values of the operational and environmental conditions that are representative of these factors’ values distributions.

Similar to the feature selection, the self-space approximation is strongly case specific. Testing, with the support of a critical analysis of similar case studies in the literature allows the identification of the exogenous factors, which affect the structural response, inducing a fluctuation in the damage-sensitive features. As introduced in Section 2.1, depending on the actual condition, details, material and geometrical properties of the structural system, different phenomena are expected to influence its behaviour. Given the specific environment of the structure and its conditions of use, it is possible to distinguish whether these phenomena are likely to happen and, eventually, discriminate the involved exogenous factors. Finally, once the exogenous factors are identified, their expected range of variation and their potential trend must be predicted. Due to the diffusion of weather stations and to the availability of historical climate data, it is possible to have not only a good estimation of the expected limits of variation for the environmental factors in a certain geographic area, but also a good forecast of their future values. Similarly, the frequency and intensity range of the operational conditions affecting the structural vibration properties can be, in many cases, predicted. Thus, even without deploying a dedicated monitoring system on the investigated structure to track the environmental and operational factors, one can use nearby existing monitoring stations to produce a preliminary estimate of their values and to plan the acquisitions in order to be as representative as possible of the expected range of the exogenous conditions.

The acquisition itself is performed in the second stage of the approximation strategy. When the self-region of the feature space is sufficiently compact and convex, and the expected range of the feature value variation is known, it is easy to achieve a good approximation of S only based on a few points. However, the fluctuation of a structural feature is rarely known in advance. The approximation strategy is thus based on the assumption that, due to the correlation between EOVs and system’s response, records that are collected from the system in extreme exogenous conditions should produce extreme values of the features distribution. Thus, sampling the features by performing acquisitions under different operational and environmental conditions that are as representative as possible of the EOVs range, is likely to produce a sufficiently unbiased sampling of the structural features. A second challenging problem, related to the self-space approximation, is the definition of a reliable index of the quality of the sample that can be used to determine whether the preliminary monitoring produced enough information. In this regard, some measures are presented and tested in Section 3.2.1.

Two diametrical approaches for the approximation of the self-space are discussed hereafter. The first one is deterministic, whereas the second one is stochastic. The two essentially differ for the nature of the analysed variable. In the former, the goal is to use the points to create an approximate self-space as the region within the envelope of the points, irrespective of their distribution statistics. In the latter, the self-space is considered as a stochastic variable whose probability density function is investigated based on the available samples.

**Deterministic approximation**. The hypothesis underlying this approach is that the self-space corresponds to the area within the most shrunk boundary enveloping the available points, intended as pairs of measured features. In many real applications there is no clear definition of what the most shrunk boundary is. The lack of a univocal interpretation of such a notion complicates its automatization. In the present work, a built-in MATLAB function, based on the concept of alpha shapes and convex shapes, is exploited to address this aspect. Add-on algorithms aimed at the same goal are under test and development.

**Stochastic approximation**. The stochastic approximation is strictly related to the actual distribution of self-samples in real case studies. Indeed, self-points typically fall into a relatively stable range presenting dense areas of overlapping samples surrounded by sparse areas of rare samples. The stochastic approximation aims at defining the probability distribution of the full self-population based on the distribution of a training set. Only a few studies in the field of binary classification, particularly through NSAs, address this specific problem, thus some techniques have here been collected and tested. All of them define an approximation of the self-set as the area within a circle of given radius around each sample. To take into account the varying distribution, samples have a different radius, meaning that a bigger radius is set for the samples in the dense region, and a smaller radius is set for the samples distributed in the sparse region and close to the boundary.

Three approaches are compared, using the same instances analysed for the deterministic case. The first one, called the affinity method [53], is based on the affinity density of each sample a calculated as:

(3) $$\rho \left(a\right)=\frac{1}{N-1}\sum_{i=1}^{N}\frac{1}{d\left(a,x^{i}\right)},\;\left(x^{i}\neq a\right),$$

where $d\left(a,x^{i}\right)$ is the Euclidean distance between the sample a and the other $\left(N-1\right)$ samples. In this case, the sample radius is given by:

(4) $$r_{self}\left(a\right)=R\;\frac{\rho \left(a\right)-\rho_{min}}{\rho_{max}-\rho_{min}},$$

where R is the radius value for the maximum affinity density sample, whereas $\rho_{min}$ and $\rho_{max}$ are, respectively, the minimum and the maximum affinity density for the sample.

The second approach is called the Gaussian method [54], since the probability distribution of the samples is approximated by a Gaussian multivariate distribution. According to the central limit theorem, the distribution of the sample mean is near to a Gaussian distribution. As the number of samples increase, the mean of the samples approaches the mean of a Gaussian distribution. Once the mean of each sample’s feature and the covariance matrix are known, the joint Gaussian probability density function $pdf_{a}$ of the sample a is calculated. As for this method, the sample radius is computed as:

(5) $$r_{self}\left(a\right)=R\frac{pdf_{a}-pdf_{min}}{pdf_{max}-pdf_{min}}\;,$$

where R is the radius value for the maximum probability density sample, while $pdf_{min}$ and $pdf_{max}$ are, respectively, the minimum and the maximum probability density of the sample set.

Finally the B-NN-ASR algorithm developed by [55] is applied as a third approach. According to this method, for each self-sample, the distance to the k-th nearest neighbours is calculated and the vectors, $v_{i}$, connecting the sample to these neighbours are defined. The sample is classified as boundary or internal according to the angle between such vectors and the vector sum, $v_{sum}=\sum_{i}^{k}v_{i}$ as follows:

(6) $$ang_{i}=\frac{180{}^{\circ}}{\pi }\arccos\left(\frac{v_{i}\cdot v_{sum}}{\left\|v_{i}\|\times \|v_{sum}\right\|}\right).$$

The sample results internal if $\max\left(ang_{i}\right)\geq \alpha$, otherwise it is classified as boundary. Internal samples have a radius equal to the distance to the k/2-th nearest neighbour. A boundary sample a is moved in the direction of the closest internal neighbour b up to the point $\frac{a+b}{2}$, and its radius is defined as:

(7) $$r_{self}\left(a\right)=\frac{dis\left(a,b\right)}{2}.$$

In the present study, two different values for R and k are compared, namely R=0.1, R=0.2, k=4 and k=6. Such values are selected through a pilot study within the range of values found in the literature.

### 2.5. Combined Strategy

**Figure 4:**
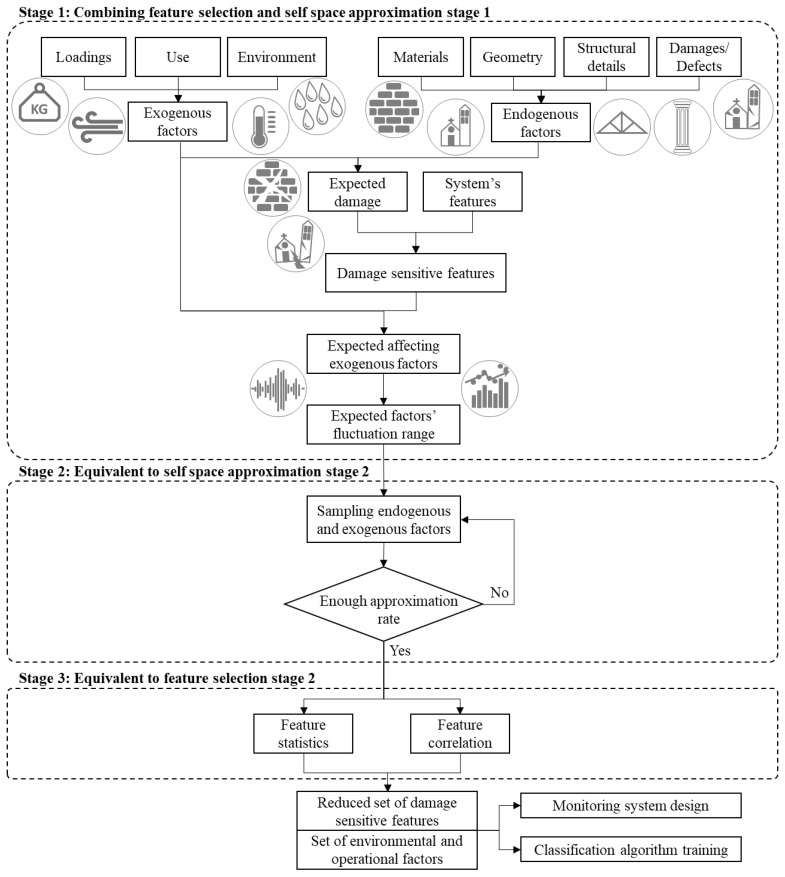
Flowchart of the combined damage detection methodology.

Feature selection and acquisition optimisation by self-space approximation are presented separately in the previous sections, since in real-world applications they are not necessarily needed at the same time. For instance, if one has to design and deploy a monitoring system for damage detection on a well-known building, feature selection might be barely addressed and minimising the samples to train the classification algorithm is the priority. On the opposite, when a large baseline dataset is already available and the asset presents a complex behaviour, selecting the most representative features, by means of numerical simulations and testing, is more important than optimising the sampling. However, the two tasks present several common activities, thus they are prone to be combined into an integrated methodology suitable for the design of a sensor network for damage detection of assets in case of very little knowledge. The flowchart depicted in Figure 4 provides a visual overview of the proposed methodology.

The first design step of the flowchart coincides with the definition of the most relevant endogenous and exogenous factors that can affect the structural behaviour in order to select the expected damage scenarios. This step collects the first stage of both strategies as they are largely overlapping.

The second stage of the self-space approximation, instead, is preparatory to the second stage of the feature selection, thus, they are presented in a sequential order.

## 3. Case Study: The Church of ‘Santa Maria de Belém’ in Lisbon

The Monastery of Jerónimos with the Church of ‘Santa Maria de Belém’, sited in Lisbon, is most likely one of the most relevant monuments of the Portuguese architectural heritage and a notable example of the Late Gothic Manueline style. It was built in phases, from 1499, undergoing several interventions and modifications over time, especially in the 18th and 19th centuries. It is worth noting that the present methodology is tested on the case study ex-post, since inspections, numerical analyses, and monitoring were performed before, hence they were not planned by strictly following the methodology proposed herein. Nonetheless, the large amount of information available on the monument is a priceless validating dataset for testing the approach and its numerical features. Until the recent investigations carried out by the University of Minho [10,11], the information at disposal about the material properties and some of the structural details was very scarce. The vulnerability of this monument, emerged after in-depth testing campaigns and numerical analyses, combined with its exposure to a medium-high seismic hazard, suggested for the deployment of a long-term monitoring system in the main nave. The data used in this study is composed of 788 records collected through this monitoring system, measured in 255 different days between 25 June 2005 and 28 September 2009 (Table 1).

A reliable estimation of the first, third, and fourth natural frequencies was possible throughout the investigated period. This dataset is the result of a data cleansing process with the rejection of corrupted measurements, thus the number of records for the different features is not always the same. For an extensive description of the inspection and monitoring campaign carried out in the Church of Jerónimos, the reader is referred to [10,11]. Hereafter, only the baseline information necessary to pursue the objectives of this work will be considered.

### 3.1. Selection of Representative Features

The two-stage feature selection procedure described in Section 2.3 is here adopted to select the most meaningful QoI of the church for damage detection purposes. As previously mentioned, the first stage requires a comprehension of the specific risks associated with the structure under investigation. The church is located in one of the areas with the highest seismic hazard of Portugal. Indeed, the country is situated near the Eurasia-Africa plate boundary, opposite the Atlantic Ocean. Even though the structure was able to withstand the great Lisbon earthquake of November 1755, the following shakes of December 1756 led to the collapse of one of the columns and, consequently, to the ruin of part of the nave. The columns of the nave are essential to support the curved vault that springs from the two longitudinal walls and whose span is reduced through their large fan capitals. Yet, these columns are very slender, featuring a free height of 16 m and a radius of just 1.04 m, which slightly increases up to 1.88 m at the nave-transept intersection. During the surveys, transversal deformations of the walls of around 6 cm and out-of-plumbness of the columns ranging between 5 and 18 cm were measured. Moreover, cracks and signs of material detachment were found in the vault and in the columns. Non-destructive tests allowed to determine the section of both the vault (stone slab of 8–10 cm, laying on stone ribs and covered with a mortar layer of variable thickness) and the columns. The latter are either monolithic or built with more limestone blocks (up to two-three blocks in the nave and four in the transept), but cramp irons between the blocks were not found.

**Figure 5:**
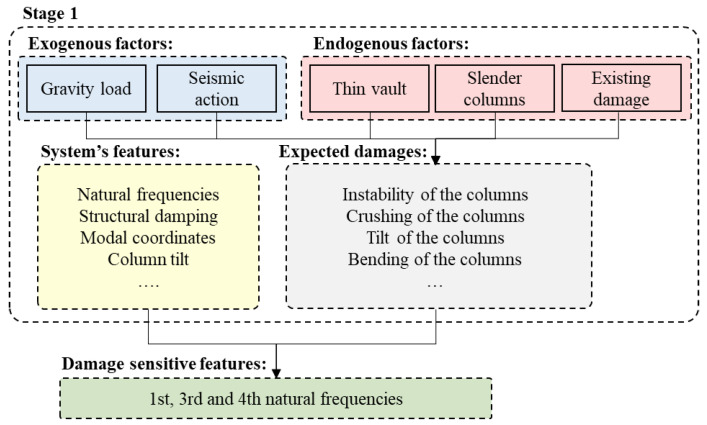
Summary of the first stage of the feature selection strategy.

The various diagnostic investigations carried out in the church over years identified the vault and the columns as the most vulnerable elements. Numerical analyses performed through finite element models with different levels of refinement proved that the activation of the most probable collapse mechanisms is indeed associated with the failure of these elements. Furthermore, it emerged that the columns’ bearing capacity plays a fundamental role not only against horizontal forces, but also in terms of building safety under gravity loads [10]. From a dynamic standpoint, it was shown that the structural behaviour of the church in the low frequency range is governed by the local modes of the slender columns. Their effect is also evident in the global response of the structure for higher frequency levels, as confirmed by the outcome of the Ambient Vibration Tests (AVTs) performed over 30 points distributed across the lateral walls, the top of the columns and the vault key. Since the deflection of the columns appears to be well caught in the analysis of the modal response of the church, it is evident that their long-term behaviour can be tracked by a vibration-based monitoring. Therefore, in this first stage, natural frequencies are identified as the most appropriate damage-sensitive features. For the sake of completeness, following the general flowchart presented in Figure 2, a detailed scheme of the first stage of the feature selection strategy is reported in Figure 5. The QoI identified for the case study object of analysis are highlighted together with the most relevant operational, loading, and environmental factors affecting the structural behaviour.

**Figure 6:**
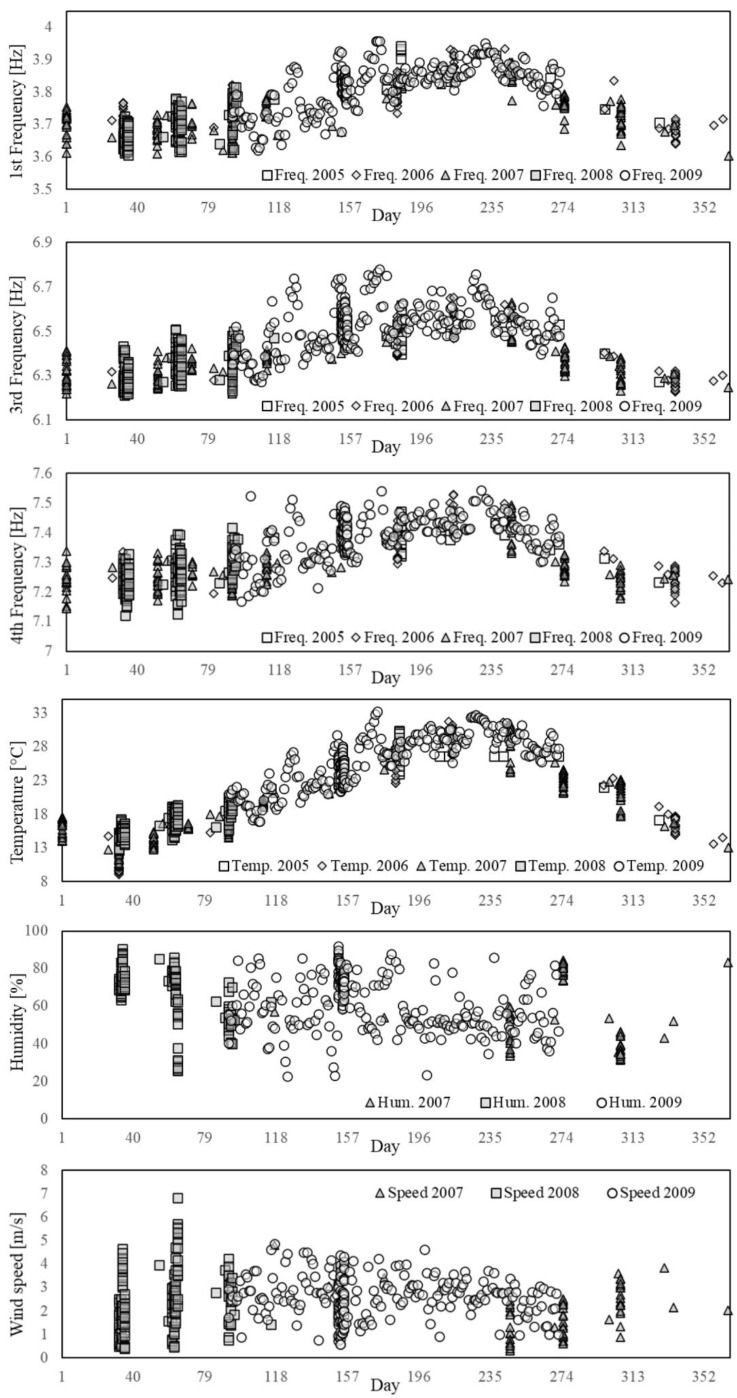
Seasonal trends of the monitored features.

**Figure 7:**
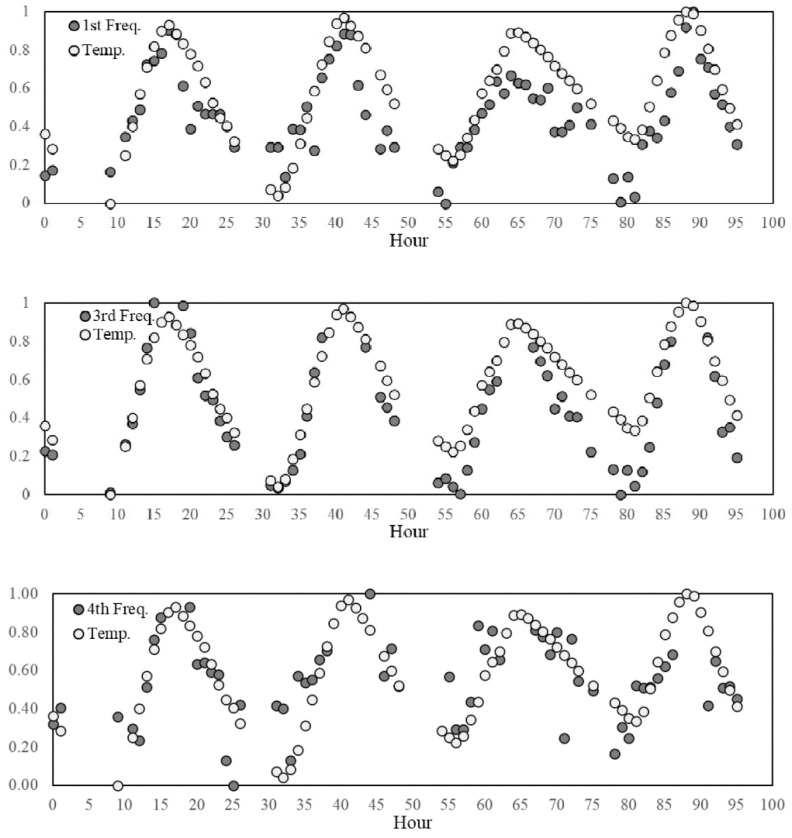
Example of the daily trend between 1 March and 4 March 2008.

In the second stage, the features variation and their correlation with the environmental factors are investigated. The influence of the thermo-hygrometric parameters on the structural dynamic properties can be qualitatively evaluated by visualising the seasonal trends illustrated in Figure 6. As it can be observed, the trends of temperature and natural frequencies present clear similarities, although a higher dispersion for the frequency samples is noted as compared to the smooth variation featured by the temperature values. This is also evident by zooming in on the daily trend, as shown by the examples reported in Figure 7 where the features are normalised to the [0,1] interval defining the unitary feature space. Such a behaviour might be due to the influence of other exogenous factors on the frequency values, such as humidity and wind speed even though these parameters do not follow a clear seasonal trend (Figure 6).

**Figure 8:**
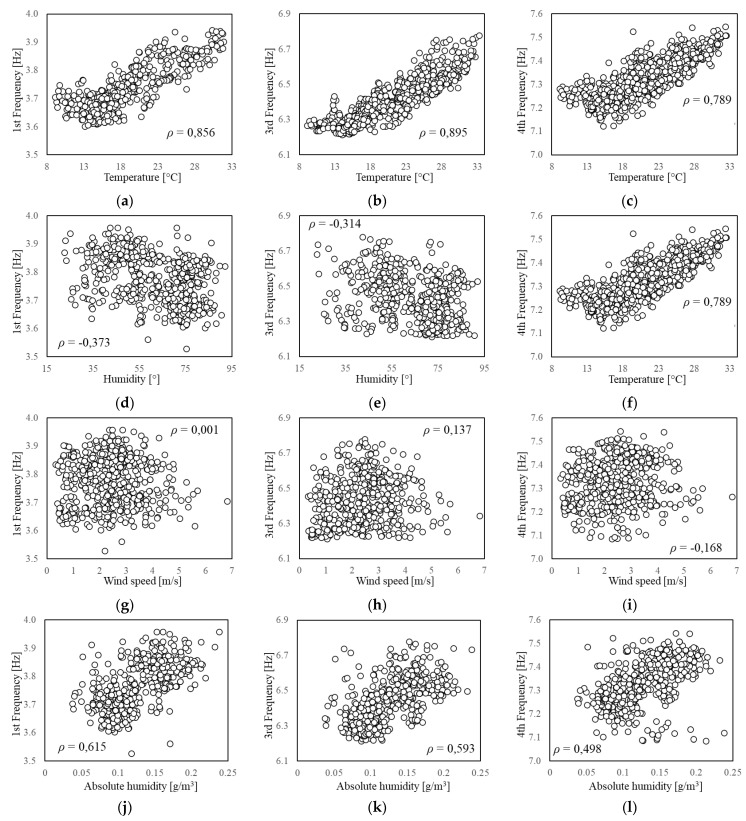
Scatterplot. Correlation between environmental factors and natural frequencies.

As scatter plots offer a better insight into the behaviour of each feature and their correlation, all the pairwise combinations of features are displayed in Figure 8. The correlation is quantified through the Pearson coefficient, ρ, which is reported in the same plots. Since the goal of the feature selection is the identification of pairs of properties to be jointly monitored, the first check consists in evaluating whether or not each one of the factors, by itself, is able to explain the frequency trend. The coefficients reveal a very slight correlation between frequencies and humidity (Figure 8d–f), and a negligible correlation between frequencies and wind speed (Figure 8g–i), meaning that the stress level induced by the wind in the structure is very low. Moreover, the Pearson coefficient confirms the good correlation between frequencies and temperature (Figure 8a–c). Finally, for the sake of completeness, the two factors with the largest correlation (e.g., air temperature T and relative humidity RH) are combined into a single parameter, namely the absolute humidity AH, according to the following formula:

(8) $$AH=\frac{6.112\cdot \exp\left[\left(17.67\times T\right)/\left(T+243.5\right)\right]RH\cdot 18.02}{\left(273.15+T\right)\cdot 8.314}\;\;(g/m^{3}),$$

and its correlation with the frequency values is also investigated. This parameter has been used to analyse monitoring data from adobe historical buildings (e.g., [32,33]), whose dynamic response demonstrated to be strongly affected by humidity. However, for the present case study, the T-AH combination does not produce a better correlation (Figure 8j–l).

**Figure 9:**
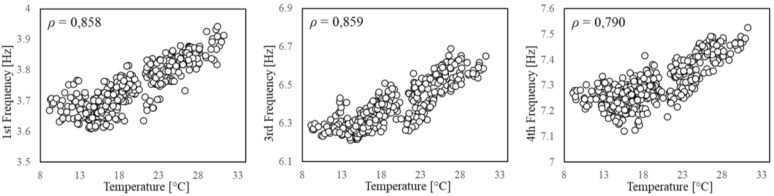
Scatterplot. One-hour lag between temperature and frequency.

**Figure 10:**
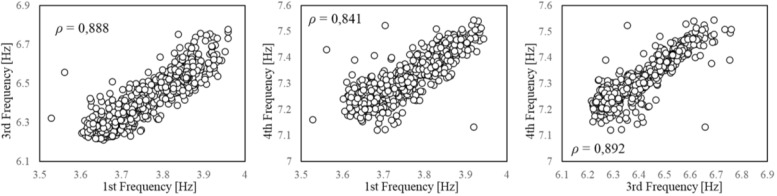
Scatterplot. Pairwise comparison between natural frequencies.

It is well-known that, if measured in distinct structural parts, environmental factors can present different variability ranges and time lags in their peaks due to the material characteristics and the local conditions and exposure of the different locations. This time lag can also interest the EOVs effect on the system vibrational properties [33,60,61]. Indeed, EOVs within a single element or among different elements are rather non-uniform and non-linear, especially in large-scale structures [12,35,62]. A recommended solution would be to perform the feature selection considering different measurement points and testing the correlation with properly averaged values. However, for the sake of simplicity, in the present study, only a single measurement point for the environmental factors is adopted, and two possible alternatives are considered. First, the degree of correlation between natural frequencies and temperature is estimated with a time shift of one hour, since the sampling rate of the measurements allows to consider only hourly lags, but this produces no further improvement in the Pearson coefficient (Figure 9). Then, the correlation is estimated only between frequencies. The pairwise comparison of these global dynamic properties shows that a very high correlation exists (Figure 10). A damage detection performed on a feature space composed of two natural frequencies might be even more effective, taking advantage of their different sensitivities to possible damage scenarios. However, such a feature space lacks the control on the exogenous factors’ variability, hence it is essential to sample the QoI for the training in order to guarantee the diversity of the environmental conditions.

In light of the foregoing, it is inferred that for the present case study different classifiers may be analysed in parallel, considering feature spaces composed by temperature and natural frequencies as well as by pairs of frequencies.

### 3.2. Approximation of the Self-Space

In the previous section, the feature selection was performed considering a large dataset. In most applications, however, one can train the damage identification algorithm based only on a few data, given the difficulties associated with extensive diagnostic investigations and monitoring campaigns. The approximation strategies tested hereafter allow to reduce the number of acquisitions required for the application of supervised learning methods by introducing some control on the QoI sampling so as to ensure that the records are sufficiently representative of the expected range of variation. Some indicators of the quality of the approximation are also defined in order to assess if the sampling provides an adequate number of points or if more acquisitions are needed. For the sake of brevity, only the analyses performed on the feature space composed by air temperature and first natural frequency are reported and discussed next. Nonetheless, the approach can be generalised to any pair of features. As per common practice in classification, especially when working with NSAs, the feature space is normalised to a unitary space.

#### 3.2.1. Deterministic Approximation

As mentioned in Section 2.4, the deterministic approximation of the self-space consists in sampling the points of the feature space belonging to the self-region and defining the area within the most shrunk boundary that envelopes them.

To analyse the performance of the approximation, it is assumed that the full self-space corresponds to the area enclosed by the enveloping boundary of the whole measured dataset (788 records). In real situations, no a priori information regarding the frequency bounds is usually available. It is also assumed that the temperature is the main exogenous factor that affects the system behaviour in the reference condition. Data provided by the climate archive for Lisbon (e.g., the “Instituto Português do Mar e da Atmosfera” [63]) report an average minimum in the coldest month around 8.1 °C and an average maximum in the warmest month around 27.8 °C. Extreme values recorded are 0.4 °C and 41.5 °C. The average annual maximum temperature is 20.9 °C, the average annual minimum value is 13.1 °C and the annual mean is 17 °C. Based on these data, a sampling is predefined by dividing the temperature line in three ranges (<17°, 17°–25°, and >25°) and collecting an equal amount of data in each range. Further analysis of the temperature distribution might lead to a better definition of the ranges. From each subrange, 2, 3, 6, 12, 18, 25, 33, 40, and 50 samples are drawn, resulting in a total of 6, 9, 18, 36, 54, 75, 99, 120, and 150 samples. To assess the influence of the number of subranges, different divisions are analysed, respectively, $\left\{3,4,6,9\right\}$ and $\left\{3,4,6,8\right\}$, for the case of 36 and 120 samples. The random generation of the samples simulates a real-world modal identification with the only condition that it is carried out in a pre-defined range of temperature; furthermore, 10 repetitions per each number of samples are performed. No repeated samples are admitted in the same set.

All these instances are compared against the exact self-space in terms of similarity, intersection area and amount of covered non-self-space. The similarity is expressed as:

(9) $$Similarity=1-\frac{Union-Intersection}{Union}\;.$$

The first two metrics (similarity and intersection area) are an estimate of the percentage of true negatives correctly classified, whereas the latter (covered non-self-space) is an estimate of the percentage of false negative errors. False negatives are samples belonging to a damaged state but classified as normal, being therefore considered the worst class of errors in damage detection. It is worth noting that the self-space approximation is only valid in the temperature range of the samples and the model beyond such bounds cannot be inferred. However, the aim is not to assess the quality of the model itself, but the amount of the global self-space that is represented, thus each approximate area is compared against the full self-space.

**Figure 11:**
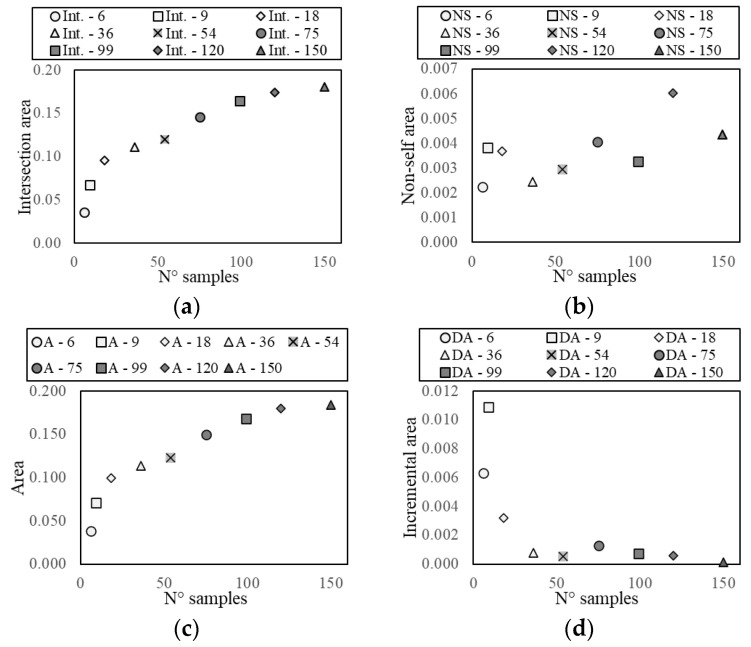
Trend of the intersection area (**a**), covered non-self-area (**b**), approximate area (**c**), and normalised incremental area (**d**), for different number of samples.

As expected, the results of the deterministic approximation, reported in Table 2, show that an increasing number of samples provides higher similarity and intersection area, but also a larger covered non-self-area, even though this value has a scattered distribution, affected by the number and the randomness of the sampling (Figure 11b). For a large sample set, the non-self-coverage is due to the higher probability of sampling boundary points, meaning that the approximate area does not fall completely inside the real one, since this is not perfectly convex. For a small sample set, the number of points used to define the boundary might be insufficient to describe the non-convexity of the self-space. Therefore, this parameter cannot be controlled a priori and it is completely problem-specific.

**Figure 12:**
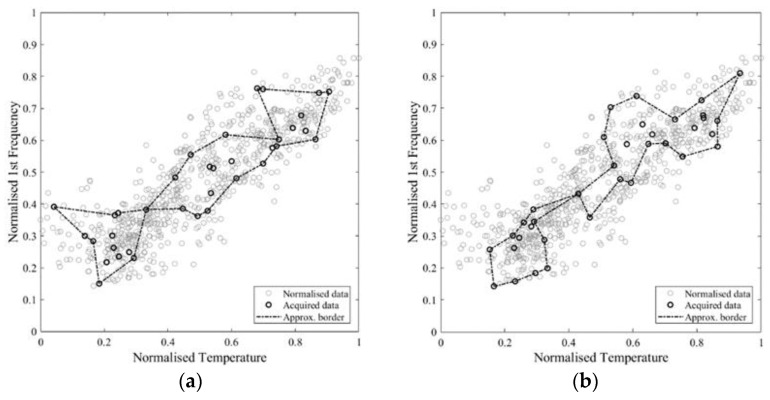
Approximate area based on a total of 36 points: (**a**) repetition 1 out of 10; (**b**) repetition 2 out of 10.

**Figure 13:**
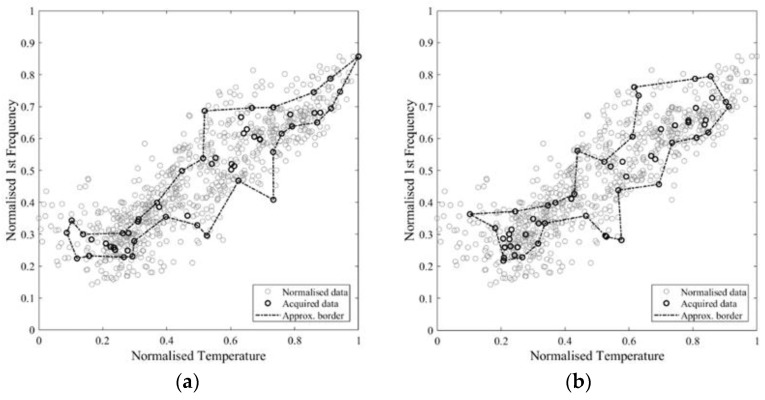
Approximate area based on a total of 54 points: (**a**) repetition 1 out of 10; (**b**) repetition 2 out of 10.

**Figure 14:**
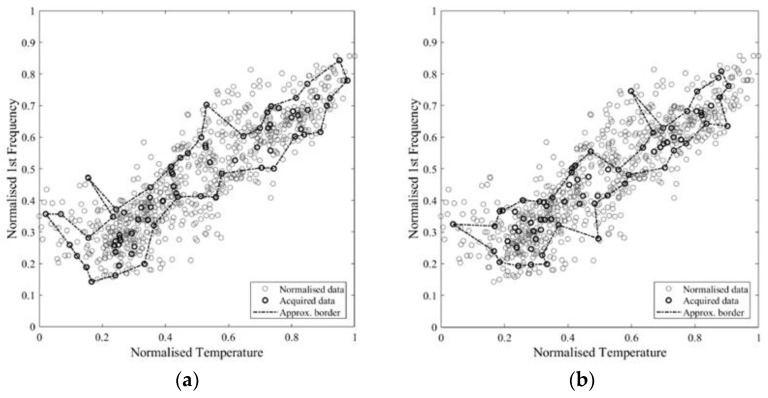
Approximate area based on a total of 75 points: (**a**) repetition 1 out of 10; (**b**) repetition 2 out of 10.

The intersection area values show a logarithmic tendency (Figure 11a). The reduction of tangent slope cannot be used directly as a criterion to define an acceptable number of samples, since it requires the a priori knowledge of the real self-area. Nonetheless, a similar trend appears in the approximate area plot and in the normalised incremental area plot (Figure 11c,d), where this latter value is equal to the area growth due to the addition of samples divided by the number of added samples. Therefore, these parameters can be used to define an acceptable number of acquisitions, which, for the specific case, is in the range 36–75. In Figure 12, Figure 13 and Figure 14, some examples of the area approximated by 36, 54, and 75 samples are reported, showing the outcome of 2 out of the 10 repetitions.

**Figure 15:**
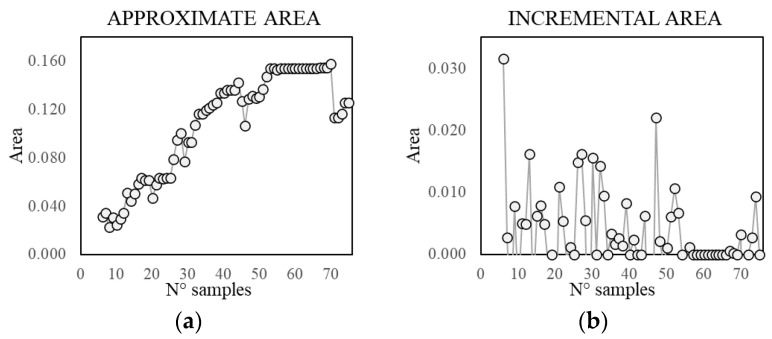
Trend of the approximate area (**a**) and normalised incremental area (**b**). For a different number of samples, a new single record was added to the previous set at each step.

**Figure 16:**
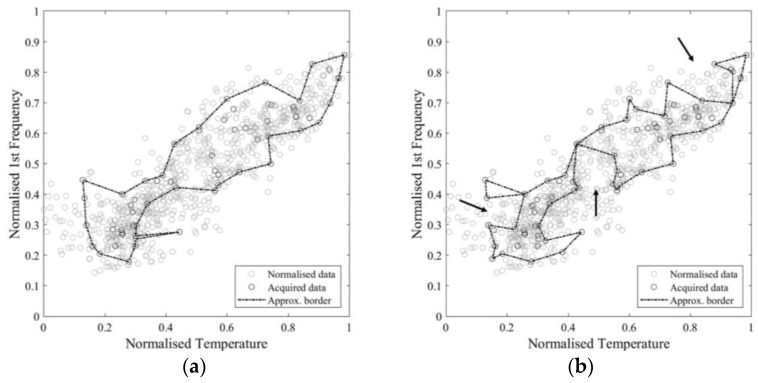
Approximate area provided by 54 (**a**) and 67 (**b**) samples.

In order to have a better insight into the sampling reduction, a single monitoring instance is simulated, starting from an initial set of 6 samples and adding 1 record per time up to 75. The resulting values of approximate area and normalised increment are reported in Figure 15. The general trend confirms the results in terms of averaged values over more repetitions as presented above. Sudden drops in the area value emerge, due to an excessive shrinkage of the boundary caused by the automatic procedure. In Figure 16, an example of such phenomenon, moving from 54 to 67 samples, is pointed out using arrows. Such an issue can be easily solved by postprocessing the automatic identified area.

**Figure 17:**
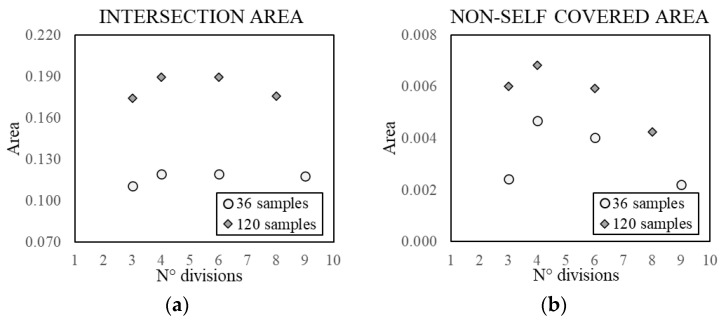
Trend of the intersection area (**a**) and non-self-covered area (**b**), for a different number of divisions and constant data samples.

The influence of the number of divisions has been analysed considering different subranges (Figure 17). The underlying idea of the division itself is to guarantee an adequate distribution of the samples along the temperature line, especially for a small dataset. Increasing the number of divisions is unlikely to improve the performance for more than four divisions in terms of approximate self-area. This phenomenon is most likely due to a forced sampling in areas of the 2D space with less interest, leaving a fewer number of samples for the dense area. Nonetheless, increasing the divisions reduces the non-self-covered area, namely the risk of false negative errors.

**Figure 18:**
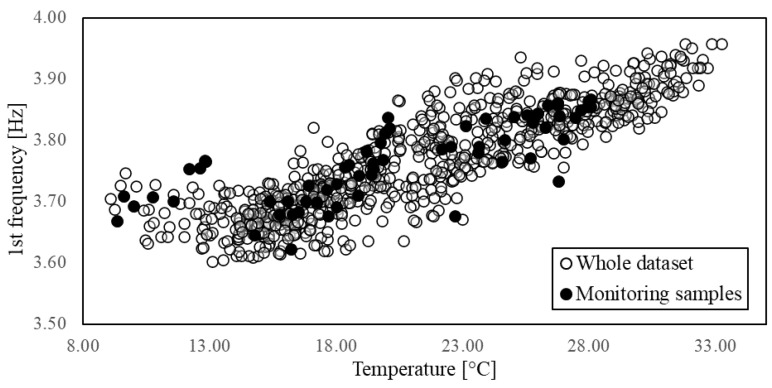
Self-dataset and monitored data campaign.

**Figure 19:**
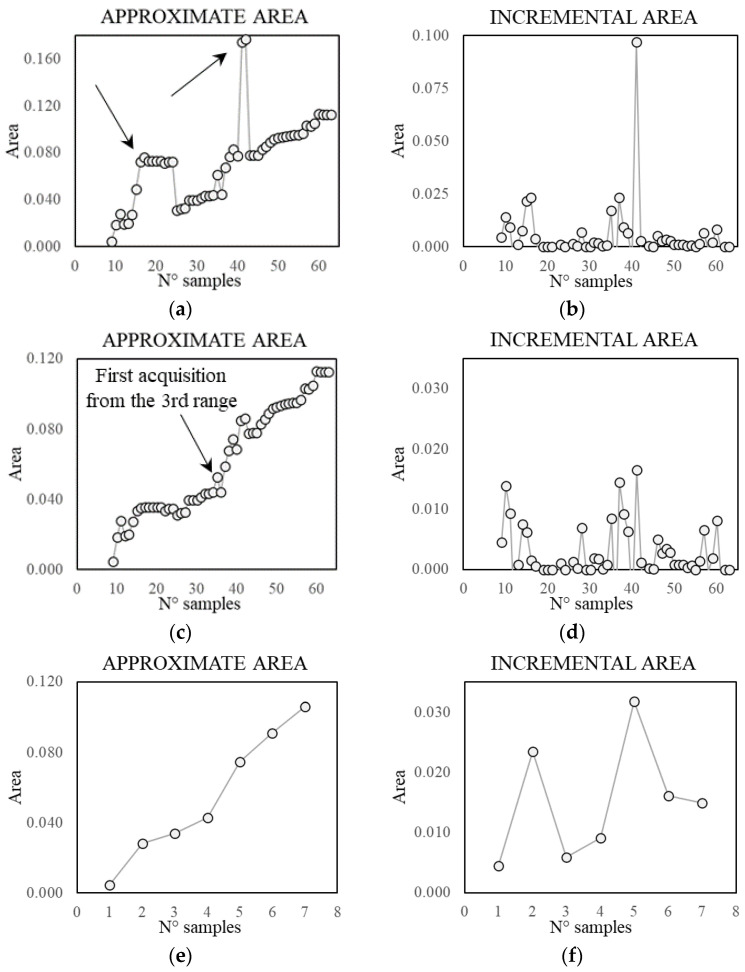
Trends of the approximate area and incremental area considering the automatic procedure (**a**,**b**), manual correction of some approximation (**c**,**d**), and daily average (**e**,**f**).

Finally, based on the available data, a possible plan for data collection is outlined. The aim is to identify 60 records, 20 for each subrange of temperature. The closest instance consists of 63 samples almost evenly distributed (18–27–18) in the 3 temperature subranges previously defined (Figure 18, Table 3). The 63 samples are collected in 7 days with hourly acquisitions from 9 a.m. to 5 p.m. between 1 February and 1 July. Since the monitoring is only simulated based on the existing dataset, the days reported do not belong to the same year. However, they are indicative of the actual temperature irrespective of the year. The trends of the approximate area and of the normalised incremental area are reported in Figure 19. As seen in the case of Figure 15, the automatic procedure yields sudden variations in the trends (Figure 19a,b). Thus, a manual modification of the approximate area is performed, achieving a smoother trend (Figure 19c,d). The trend allows to identify a small jump first due to the acquisitions collected from the second range of the temperature, during the second day. A second significant jump appears when acquisitions from the third range are added. After each jump the incremental area reduces significantly as well as the tangent slope of the approximate area. Finally, the average daily trend is introduced (Figure 19e,f) as a condensed index of the approximation quality. The average daily incremental area, in particular, is able to capture more clearly than the hourly one the sudden growth due to samples from the second and third range and the following reduction in the increment when an acceptable approximation is reached.

#### 3.2.2. Stochastic Approximation

The three stochastic approximation algorithms presented in Section 2.4 are applied and compared hereafter. Due to the characteristics of the sample set, for the B-NN-ASR, the value of α, recommended to be equal to 150° [55], is here reduced to 110° to decrease the number of points wrongly classified as external.

**Figure 20:**
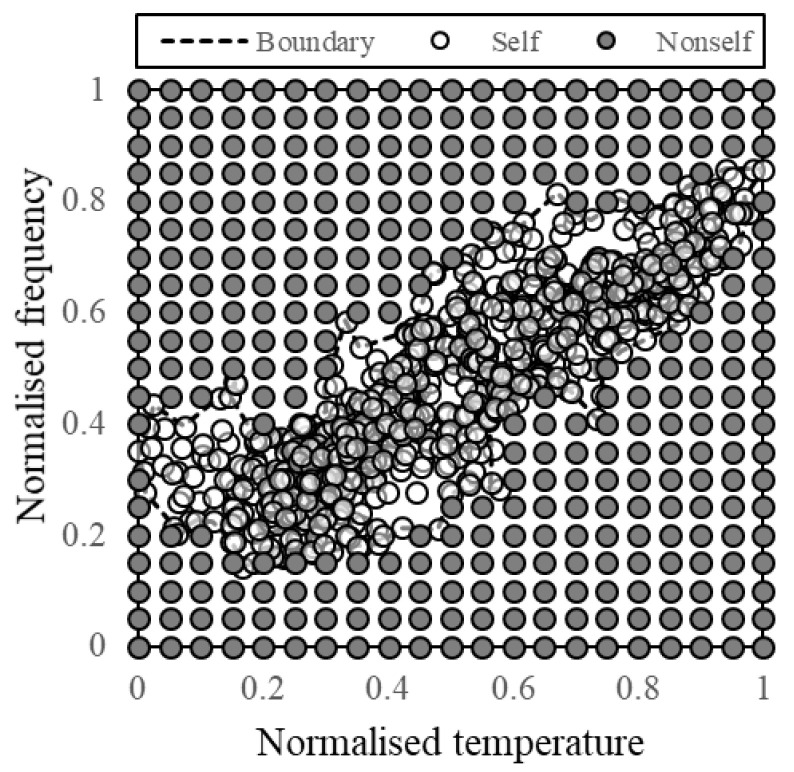
Full self-set with its boundary and artificial non-self-set used for performance assessment.

Their performance is evaluated in terms of self-coverage, SC, and non-self-coverage, NSC. Namely, given the full set of 788 self-samples and a set of 326 artificial non-self-elements (Figure 20), the coverage corresponds to the number of samples falling within the circles approximating the corresponding set:

(10) $$SC=\frac{N{}^{\circ}\;of\;self\;samples\;covered\;by\;the\;approximate\;self\;set\;}{Total\;N{}^{\circ}\;of\;self\;samples}\;NSC=\frac{N{}^{\circ}\;of\;nonself\;samples\;covered\;by\;the\;approximate\;self\;set\;}{Total\;N{}^{\circ}\;of\;nonself\;samples}$$

The two aforementioned metrics are chosen because, in this case, the circles present a large overlapping area, and a direct estimation of the covered self-space is not as straightforward as in the deterministic approximation.

**Figure 21:**
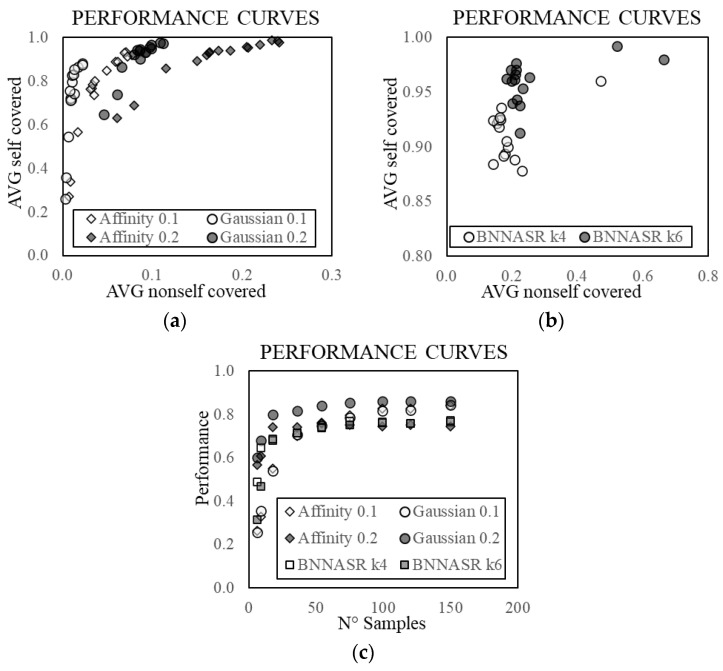
Performance curves of the different stochastic methods considering different sample sets, in terms of non-self and self-coverage (**a**,**b**). Plot of the performance (analogous to J statistic) against the number of samples (**c**).

The obtained SC and NSC are reported in Table 4. The results are also represented in Figure 21 where the values of the SC are plotted against those of the NSC as points of a performance curve for each algorithm. Based on the similarity between the receiver operating characteristic (ROC) curve and the performance curve, a possible single metric to estimate the quality of the approximation is defined, drawing inspiration from the well-known Younden’s J statistic [64]. The metric ranks each point, namely a combination of algorithm and number of samples, giving equal weight to NSC and SC. This metric corresponds to the vertical distance from the point to the diagonal line (namely the difference SC–NSC). In Table 4, for each performance curve, the point with the highest distance is reported in bold and the best overall performance is underlined. Although most of the algorithm instances reach the best performance for 120 samples, the metric values grow quickly up to around 36 samples, then the trend becomes almost constant for the increasing number of samples (Figure 21c). This result is consistent with the outcome of the deterministic approximation (see Figure 11).

As for the affinity and the Gaussian approaches, a tendency to increase both the self- and the non-self-coverages is noted as the number of samples increases. Given the same R, the Gaussian method outperforms the affinity, presenting a lower non-self-coverage for the same self-coverage, thus being less prone to false negative errors. However, to achieve the same self-coverage, the Gaussian approach requires more samples, especially for a smaller R. As expected, when a larger radius R is adopted, less samples are required to achieve a good coverage, however, the covered non-self-area quickly becomes significant. A smaller radius, even if more conservative, leads to the presence of holes in between the approximate self-areas, which might induce false positive errors. This phenomenon can be overcome by using the deterministic approximation since all the area within the samples is considered as belonging to the self.

**Figure 22:**
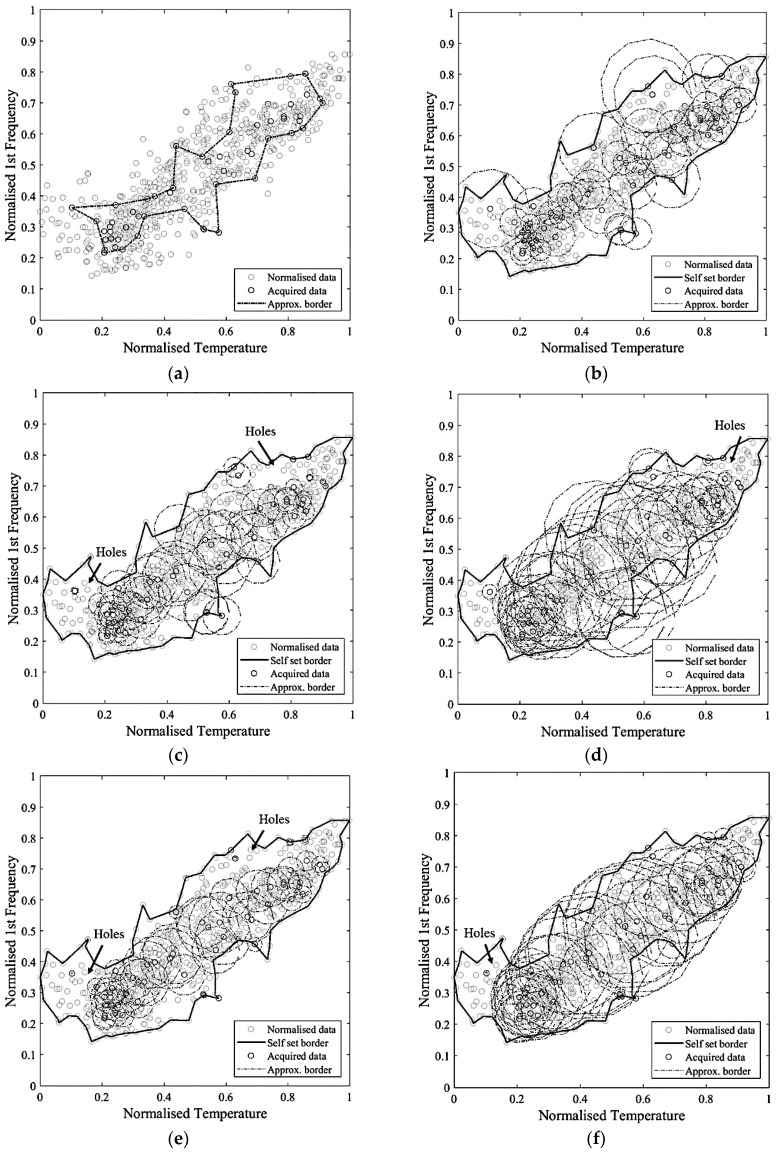
54-point approximate areas: (**a**) deterministic approximation; (**b**) B-NN-ASR k4; (**c**) affinity 0.1; (**d**) affinity 0.2; (**e**) Gaussian 0.1; (**f**) Gaussian 0.2.

Among the three methodologies, the B-NN-ASR algorithm showed a completely different behaviour, proving to be less effective. Due to the characteristics of the training set, the algorithm fails to identify the inner samples and, consequently, the boundary samples are not moved toward an internal neighbour. For the same reason, the size of the holes within the approximate self-space is much lower, but the non-self-area covered is too large. Such results can be qualitatively seen in Figure 22, where the approximate self-set, falling within the circles with variable radius around the acquired data, is compared with the full self-set that corresponds to the area enclosed by the enveloping boundary of the whole measured dataset (788 records).

## 4. Discussion

The case study of the Church of ‘Santa Maria de Belém’, in Lisbon, has been explored in this work to separately address two crucial aspects of the damage detection task: feature selection and self-space approximation. Both the strategies can be combined into an integrate preliminary step based on long-term monitoring and classification algorithm.

The process flow dissected in the previous sections can result in being beneficial not only to technicians and the scientific community operating in the field of SHM and damage identification, but also to the stakeholders and facility managers interested in investing in SHM-based approaches as supporting tools for decision making.

Once the most relevant endogenous and exogenous factors that can affect the structural behaviour are identified, critical damage scenarios can be defined. As for the present case study, past analyses and diagnostic investigations identified the columns and the thin vault of the church as the most vulnerable elements of the ensemble, as well as the structural elements mostly affecting the dynamic response of the system in the low frequency range. Thus, the natural frequencies of the first vibration modes of the church were considered as damage-sensitive features, as their value is expected to change sensibly in the case of variation in the column’s mechanical properties. The feature selection stage can be carried out based on common techniques for the structural assessment of existing buildings, thus, it does not involve extra costs for asset owners or facility managers. Information regarding the exogenous factors is also easily accessible in most of the cases due to the well-studied historical seismicity and the existence of national networks of sensors to record environmental factors (e.g., temperature and humidity) and extreme events (e.g., earthquake). A dedicated sensor network for the exogenous factors can be deployed to further refine the accuracy and the level of knowledge of the local conditions at the building or element scale, but it is not strictly required at this stage, and punctual measurements during the inspections can be easily performed. For the present case study, the vulnerability and the condition state of the structure were evaluated through in-situ surveys supported by radar investigations, opening of inspection windows, bore-drilling, chemical analysis of the materials, and operational modal analyses. Moreover, numerical simulations were performed by means of simplified models of the whole building as well as refined models of single portions of it; the results were verified through simple hand calculations [10]. Carrying out an in-depth inspection and diagnosis phase is the basic requirement for the appropriate design of a long-term monitoring system for damage detection, and it is essential to support the safety assessment of the building in its actual condition and to drive the decision-making procedures.

When aiming at the long-term damage detection, the self-space must be approximated as best as possible by the optimising number and timing of the acquisitions. As for the analysed structure, the preliminary monitoring was planned by pre-defining the days for the acquisition according to the range of variability of the temperature in the area of Lisbon, as provided by climate archives and weather forecast, since this parameter was expected to be the most influential on the dynamic behaviour of the church. For the acquisition, at least 60 samples—20 for each temperature sub-range—were considered necessary for a good representation of the featured behaviour. Nonetheless, the approximation and the monitoring are performed simultaneously, hence the approximation performance can be assessed through ad-hoc metrics that do not require any information regarding the whole dataset. If needed, more acquisitions can be demanded.

Several strategies, either deterministic or stochastic, can be used to carry out the self-space approximation stage. As shown, a direct comparison between these techniques is hardly possible, due to the differences in the functioning and in the region produced. With respect to the deterministic counterpart, the stochastic approximate space might present holes within the enveloping boundary of the samples and cover part of the feature space outside of it. Since the stochastic methods are strongly related to the mode of operation of the NSAs, they are preferable if used to prepare the training set of such algorithms. For the present case study, among the applied stochastic techniques, the Gaussian with a radius equal to 0.2 produced the best performance. The deterministic approach, on the other hand, is likely the easiest to be generalised to support the training of any damage detection algorithm. Nonetheless, a combined use of deterministic and Gaussian strategy is found to be beneficial, since it allows to assess the approximation by using different metrics (e.g., normalised incremental area and approximate area for the deterministic approach, optimum point of the performance curve for the stochastic approach); moreover, a comparison of the resulting boundaries allows a critical post-processing, for instance assessing if the deterministic approximation can be extended outside the boundary or must be reduced according to the holes found in the stochastic approximation. During this stage, a continuous monitoring system is not necessary, and this is extremely advantageous because it does not require the installation of permanent sensors on a building. Even if the costs due to deployment and dismantling of temporary sensors for each acquisition day might be significant, the time requirement for the post-processing is strongly reduced, since a very limited number of significant records is analysed. Besides, a reduced sample set implies an easier data storage. On the opposite, continuous preliminary monitoring requires the system to automatise the acquisition and transmission of data to a remote server and needs to maintain a large number of sensors occupied for a long time. In terms of data overload, these aspects can hinder the smoothness of the process, especially considering that the preliminary acquisition entails many more measurement points than ordinary long-term monitoring campaigns, as the system behaviour is unknown and must be properly investigated. Non-continuous measurements might allow the stakeholders to simultaneously perform the preliminary monitoring stage over more structures (e.g., within a six-month time horizon) even with a limited set of sensors, by alternating the days of acquisitions.

**Figure 23:**
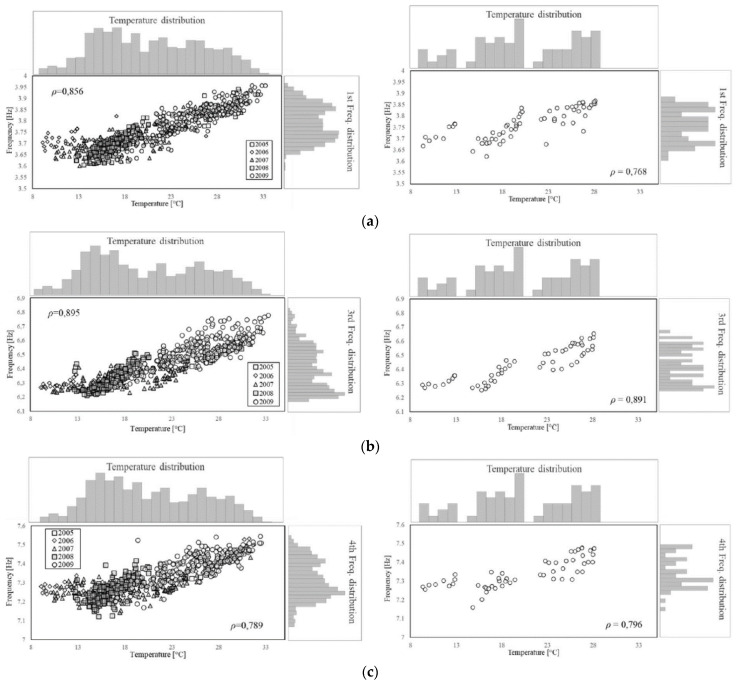
Overview of the data samples. Comparison between the whole dataset and the simulated monitoring campaign with reduced samples for the first frequency (**a**), third frequency (**b**) and fourth frequency (**c**).

In the third stage of the combined methodology, the sample set produced by the preliminary monitoring is used to identify the most representative damage-sensitive QoI for the long term monitoring. This task depends on the functioning of the damage detection algorithm. In this work, the authors referred to a recently developed version of the NSA which classifies the condition of the system within a two-dimensional feature space. Thus, the correlation between the available features was investigated to define pairs suitable to form feature spaces for the damage detection. To this end, a preliminary monitoring consisting of 63 acquisitions, from 7 days of non-continuous monitoring, between 1 February and 1 July, was simulated (see Table 3). To demonstrate that the reduced sampling is adequately representative of the whole dataset, thereby proving the effectiveness of the self-space approximation, Figure 23 shows a qualitative comparison between the whole population and the monitored data. As for the latter, the Pearson coefficient is highlighted. Table 5 reports the statistics for both sets.

It is worth noting that, even though the distribution of the monitored data is not completely representative of the population’s distribution, the Pearson coefficient values are very close, suggesting that the feature selection can be performed on reduced datasets, leading to similar results. The differences in the distribution are likely affected by a lack of samples in the highest range of temperature. The maximum value of the population is indeed 5 °C greater as compared to the monitored data. This lack might affect both the interquartile distance, which is smaller for monitored data, and the skewness, which becomes negative since the right tail is missing. Nonetheless, the measures of central tendency are properly approximated. As for the natural frequencies, it is noted that the extreme values are missing even though upper and lower ranges, which are cut off by the sampling, are comparable. Similar to the temperature, the main difference is in the skewness.

For the sake of brevity, only the correlation between frequencies and temperature is discussed here. Still, the same approach was followed to analyse the correlation between pairs of frequencies and between them and other environmental factors.

Numerical simulations can be also beneficial for the analysis of the effective damage-sensitivity of the system features to support their selection. By using advanced numerical models, the damage scenarios can be simulated together with the effect of other exogenous factors, allowing to explore the changes induced in the system response. Despite the advantages, it is important to bear in mind that this approach might fail in representing all of the most critical scenarios or in simulating them correctly, leading to a biased selection of the damage-sensitive features. Moreover, numerical simulations can be time consuming and computationally expensive, bringing on higher costs. Finally, it is stressed that whenever a specific analysis of the features’ damage-sensitivity is not performed, the system condition can still be successfully assessed, employing more classifiers in parallel and working on different well-correlated feature spaces.

## 5. Conclusions

This paper discussed a methodology to support SHM practitioners involved in the damage detection of civil engineering systems, especially historical and monumental buildings, in the definition of the most significant features/state indicators to be monitored, and in the optimisation of the number of preliminary acquisitions necessary to drive this selection. First, these two tasks were presented separately as they are not always performed simultaneously on the same asset. Then, a combination of the two tasks was described through a single workflow that merged the common activities and reorganised the specific ones. These preliminary activities are also the common code’s requirement to structural evaluation, making SHM easy to frame within existing building assessment.

It was shown that the feature selection process is extremely case-specific, depending on the system components, on the surrounding environment as well as on the conditions of use that might affect the structural response. A relevant case study was presented to provide a deep insight in this regard. Moreover, as the feature selection must be tailored to the characteristics of the employed damage detection algorithm, a Negative Selection Algorithm based methodology, well-known by the authors, was used in the present work, though the lessons learnt by this specific application can be easily adapted and generalised.

In what concerns the optimisation of the acquisition task, the motivations for reducing the complexity of the preliminary investigation (e.g., monitoring period, amount of data, number of permanent sensors, time and costs, etc.) were extensively discussed. In this regard, the presented methodology was proven capable to achieve a sufficient approximation of the system behaviour based on non-continuous acquisitions properly sampled over time. Different algorithms suitable for the methodology were tested and compared, and simple metrics to assess the quality of the preliminary monitoring and the resulting approximate feature distribution were presented. It was shown that such metrics do not require any previous knowledge regarding the system feature distributions and can be calculated during the execution of the monitoring to define when an acceptable level of knowledge is reached.

In conclusion, the proposed combined methodology appears to be a cost-effective strategy to investigate the behaviour of a poorly known system and to train a damage detection algorithm for its long-term assessment. The amount of data necessary for the training is very limited, making the information flow for damage detection easy to manage and interrogate. Nonetheless, the relevance of such data is guaranteed by identifying the most representative features and optimising its acquisition over time.

## Figures and Tables

**Table 1:** Overview of the collected data.

Year	N° Days	N° Samples	Month Range	T Range (°C)	f_1_ Range (Hz)	f_3_ Range (Hz)	f_4_ Range (Hz)
2005	11	24	25 June–22 November	17.19–30.39	3.705–3.943	6.27–6.781	7.09–7.524
2006	24	111	26 January–27December	9.09–31.83	3.639–3.939	6.23–6.652	7.16–7.53
2007	27	183	1 January–30 December	10.38–31.71	3.602–3.934	6.22–6.656	7.14–7.505
2008	17	221	1 February–24 April	12.68–25.95	3.605–3.936	6.21–6.51	7.12–7.476
2009	176	249	1 April–28 September	16.93–33.22	3.620–3.958	6.27–6.778	7.09–7.544

**Table 2:** Results of the deterministic approximation of the self-space.

Pt.	Div.	Area Avg.	Area St. dev.	Similarity Mean	Similarity St. Dev.	Intersection Area Mean	Intersection Area St. Dev.	Non-Self-Covered Mean	Non-Self-Covered St. Dev.
Exact self-space		0.276	–	100	–	1	–	0	–
6	3	0.071	0.032	12.7	5.0	0.036	0.064	0.0022	0.0048
9	3	0.099	0.124	23.8	8.6	0.067	0.065	0.0038	0.0062
18	3	0.113	0.090	34.2	7.5	0.096	0.053	0.0037	0.0059
36	3	0.124	0.151	39.8	11.9	0.111	0.083	0.0024	0.0027
36	4	0.123	0.110	42.4	7.3	0.119	0.111	0.0047	0.0056
36	6	0.120	0.109	42.6	6.2	0.119	0.088	0.0040	0.0034
36	9	0.123	0.122	42.3	6.3	0.118	0.132	0.0022	0.0025
54	3	0.150	0.158	43.0	6.8	0.120	0.126	0.0029	0.0035
75	3	0.168	0.205	52.0	11.0	0.146	0.194	0.0040	0.0070
99	3	0.180	0.199	59.0	8.4	0.165	0.161	0.0033	0.0032
120	3	0.196	0.212	61.7	7.0	0.174	0.155	0.0060	0.0070
120	4	0.196	0.205	67.0	5.9	0.189	0.166	0.0068	0.0053
120	6	0.180	0.200	67.3	4.7	0.190	0.193	0.0059	0.0027
120	8	0.184	0.150	62.7	5.4	0.176	0.182	0.0042	0.0036
150	3	0.038	0.181	64.2	5.6	0.180	0.157	0.0043	0.0023

**Table 3:** Designed data campaign with results. Three colours are used to distinguish the three ranges.

Day	Hour	Temp. (°C)	Freq.1 (Hz)	Freq.3 (Hz)	Freq.4 (Hz)		Day	Hour	Temp. (°C)	Freq.1 (Hz)	Freq.3 (Hz)	Freq.4 (Hz)
01/02	09:00	9.35	3.669	6.292	7.270		01/06	09:00	22.71	3.677	6.510	7.409
01/02	10:00	9.59	3.708	6.272	7.256		01/06	10:00	23.11	3.823	6.513	7.400
01/02	11:00	10.01	3.693	6.299	7.281		01/06	11:00	23.9	3.836	6.535	7.400
01/02	12:00	10.75	3.708	6.282	7.283		01/06	12:00	25.03	3.838	6.542	7.409
01/02	13:00	11.58	3.701	6.295	7.304		01/06	13:00	25.55	3.841	6.567	7.460
01/02	14:00	12.2	3.753	6.320	7.275		01/06	14:00	25.98	3.844	6.586	7.460
01/02	15:00	12.61	3.755	6.340	7.286		01/06	15:00	26.35	3.858	6.581	7.468
01/02	16:00	12.81	3.767	6.358	7.309		01/06	16:00	26.75	3.858	6.623	7.479
01/02	17:00	12.84	3.765	6.358	7.336		01/06	17:00	26.74	3.862	6.588	7.477
02/03	09:00	14.76	3.646	6.271	7.160		03/06	09:00	22.18	3.786	6.417	7.335
02/03	10:00	15.36	3.701	6.286	7.279		03/06	10:00	22.54	3.790	6.447	7.334
02/03	11:00	16.1	3.701	6.308	7.269		03/06	11:00	23.61	3.781	6.453	7.352
02/03	12:00	16.89	3.727	6.359	7.274		03/06	12:00	24.67	3.801	6.532	7.368
02/03	13:00	17.69	3.677	6.418	7.302		03/06	13:00	25.77	3.829	6.588	7.414
02/03	14:00	18.5	3.761	6.464	7.314		03/06	14:00	26.81	3.840	6.573	7.437
02/03	15:00	19.21	3.782	–	–		03/06	15:00	27.66	3.850	6.620	7.464
02/03	16:00	19.75	3.797	–	–		03/06	16:00	28.05	3.855	6.629	7.474
02/03	17:00	19.92	3.811	–	–		03/06	17:00	28.07	3.867	6.655	7.474
03/03	09:00	15.77	3.680	6.254	7.204		01/07	09:00	23.64	3.790	6.398	7.313
03/03	10:00	16.27	3.680	6.286	7.242		01/07	10:00	24.56	3.766	6.404	7.312
03/03	11:00	16.82	3.701	6.324	7.350		01/07	11:00	25.67	3.771	6.433	7.310
03/03	12:00	17.62	3.720	6.368	7.316		01/07	12:00	26.26	3.821	6.455	7.350
03/03	13:00	18.02	3.729	6.395	7.343		01/07	13:00	26.8	3.734	6.500	7.351
03/03	14:00	18.35	3.757	6.405	7.301		01/07	14:00	27	3.802	6.514	7.395
03/03	15:00	18.91	3.742	–	–		01/07	15:00	27.43	3.837	6.539	7.403
03/03	16:00	19.46	3.763	–	–		01/07	16:00	27.92	3.855	6.543	7.403
03/03	17:00	19.47	3.755	–	–		01/07	17:00	27.97	3.864	6.566	7.442
04/03	09:00	16.23	3.622	6.265	7.265							
04/03	10:00	16.52	3.683	6.284	7.261							
04/03	11:00	17.22	3.699	6.317	7.263							
04/03	12:00	18.02	3.691	6.377	7.275							
04/03	13:00	18.86	3.710	6.428	7.293							
04/03	14:00	19.39	3.744	6.459	7.309							
04/03	15:00	19.85	3.768	–	–							
04/03	16:00	20.1	3.819	–	–							
04/03	17:00	20.03	3.837	–	–							
Colour key:												
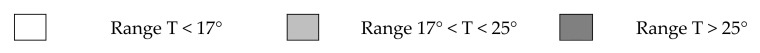												

**Table 4:** Average self-set and non-self-set coverage of the different stochastic approximations. In each column, the point with best performance is highlighted in bold. The best overall performance is underlined.

Pt.	Div.	Affinity 0.1		Affinity 0.2		Gaussian 0.1		Gaussian 0.2		B-NN-ASR k4		B-NN-ASR k6	
		SC	NSC	SC	NSC	SC	NSC	SC	NSC	SC	NSC	SC	NSC
6	3	0.269	0.007	0.629	0.060	0.259	0.002	0.649	0.046	0.960	0.471	0.980	0.664
9	3	0.337	0.009	0.688	0.080	0.359	0.004	0.740	0.061	0.878	0.231	0.992	0.521
18	3	0.567	0.017	0.857	0.115	0.547	0.006	0.866	0.066	0.888	0.206	0.913	0.224
36	3	0.737	0.035	0.891	0.150	0.716	0.009	0.903	0.086	0.894	0.179	0.938	0.224
36	4	0.765	0.033	0.930	0.163	0.722	0.008	0.934	0.092	0.900	0.186	0.953	0.233
36	6	0.762	0.031	**0.934**	**0.164**	0.713	0.009	0.932	0.092	0.892	0.173	0.944	0.215
36	9	0.785	0.033	0.941	0.174	0.742	0.013	0.949	0.099	0.905	0.182	0.963	0.252
54	3	0.800	0.036	0.919	0.160	0.758	0.008	0.922	0.079	0.884	0.141	0.940	0.201
75	3	0.846	0.049	0.940	0.187	0.798	0.010	0.940	0.086	0.922	0.154	0.961	0.208
99	3	0.887	0.059	0.954	0.207	0.826	0.010	0.944	0.083	0.924	0.158	0.960	0.198
120	3	0.889	0.061	0.957	0.205	0.832	0.012	0.947	0.086	0.918	0.161	0.966	0.209
120	4	0.918	0.077	0.977	0.241	0.864	0.017	0.965	0.099	0.936	0.166	0.977	0.212
120	6	**0.933**	**0.071**	0.984	0.240	**0.883**	**0.021**	**0.975**	**0.109**	0.925	0.164	0.970	0.213
120	8	0.931	0.069	0.987	0.233	0.874	0.022	0.972	0.112	**0.925**	**0.142**	**0.962**	**0.182**
150	3	0.913	0.072	0.965	0.220	0.856	0.013	0.957	0.097	0.927	0.163	0.970	0.197

**Table 5:** Statistics overview. Comparison between whole and reduced (simulated monitoring) datasets.

	Whole Dataset				Monitored Dataset							
	T (°C)	f_1_ (Hz)	f_3_ (Hz)	f_4_ (Hz)	T (°C)	ΔT	f_1_ (Hz)	Δf_1_	f_3_ (Hz)	Δf_3_	f_4_ (Hz)	Δf_4_
Max	33.22	3.96	6.78	7.54	28.07	−15.5%	3.87	−2.3%	6.66	−1.9%	7.48	−0.9%
Min	9.09	3.53	6.21	7.09	9.35	2.9%	3.62	2.7%	6.25	0.7%	7.16	1.1%
Mean	21.16	3.77	6.42	7.32	20.31	−4.0%	3.77	~0%	6.43	0.3%	7.34	0.4%
St. Dev.	5.64	0.09	0.13	0.10	5.42	−3.9%	0.07	−24.4%	0.12	−11.9%	0.08	−17.9%
Skewness	0.15	0.10	0.48	0.10	−0.25	−271%	−0.14	−245%	0.14	−69.9%	0.20	96.2%
Kurtosis	−0.99	−1.0	−0.7	−0.7	−0.94	−5.0%	−1.05	4.2%	−1.27	86.9%	−0.68	−1.7%
Median	20.73	3.76	6.40	7.30	19.75	−4.7%	3.77	0.1%	6.42	0.4%	7.33	0.3%
Interquartile	9.45	0.14	0.23	0.15	8.94	−5.4%	0.12	−16.9%	0.22	−4.0%	0.12	−19.7%
Max. daily range	7.14	0.20	0.26	0.27	–	–	–	–	–	–	–	–
Min. daily range	1.27	0.04	0.09	0.05	–	–	–	–	–	–	–	–

## Data Availability

Not applicable.
